# Beyond English: Considering Language and Culture in Psychological Text Analysis

**DOI:** 10.3389/fpsyg.2022.819543

**Published:** 2022-03-04

**Authors:** Dalibor Kučera, Matthias R. Mehl

**Affiliations:** ^1^Department of Psychology, Faculty of Education, University of South Bohemia in České Budějovice, České Budějovice, Czechia; ^2^Department of Psychology, College of Science, University of Arizona, Tucson, AZ, United States

**Keywords:** natural language processing, cross-language, culture, closed-vocabulary approaches, LIWC

## Abstract

The paper discusses the role of language and culture in the context of quantitative text analysis in psychological research. It reviews current automatic text analysis methods and approaches from the perspective of the unique challenges that can arise when going beyond the default English language. Special attention is paid to closed-vocabulary approaches and related methods (and Linguistic Inquiry and Word Count in particular), both from the perspective of cross-cultural research where the analytic process inherently consists of comparing phenomena across cultures and languages and the perspective of generalizability beyond the language and the cultural focus of the original investigation. We highlight the need for a more universal and flexible theoretical and methodological grounding of current research, which includes the linguistic, cultural, and situational specifics of communication, and we provide suggestions for procedures that can be implemented in future studies and facilitate psychological text analysis across languages and cultures.

## Introduction

The use of computerized text analysis as a method for obtaining information about psychological processes is usually dated to the 1960s, when the General Inquirer program was introduced (Stone et al., 1962). Since then, this field has advanced and flourished in ways that were difficult to foresee at the time. The original (word-count) approaches have been enhanced and optimized in terms of the scope and complexity of their dictionaries and methods (Eichstaedt et al., 2020), and the capacity of computers has arrived at processing very large amounts of data in no time. At the same time, extensive digital documentation and sharing, related to the growth of the information society (Duff, 2000; Fuller, 2005), have provided almost unlimited input for text analysis.

Over the last decade, Natural Language Processing (NLP) methods have effectively become an established and attractive go-to method for psychological science (Althoff et al., 2016; Pradhan et al., 2020). At present, they are developed mainly as automated systems that can understand and process texts in natural language, e.g., for conversational agents, sentiment analysis, or machine translation (Amini et al., 2019). The new techniques, employing methods of artificial intelligence, classical machine learning (ML), and deep learning methods (Magnini et al., 2020) are gradually displacing original approaches, with their eventual dominance in the field being a safe prediction (Johannßen and Biemann, 2018; Eichstaedt et al., 2020; Goldberg et al., 2020).

By implication, the field can currently be thought of as being in a transitional phase—although most cited studies in psychology are based on foundations laid with conventional computational techniques (e.g., word counting), their share is gradually decreasing in favor of more complex techniques (e.g., ML processing). This phase is crucial in many ways, not only for the (re)evaluation of existing research backgrounds and evidence but also for the development and optimization of next-generation psychological text analysis methods.

The goal of this article is to provide a critical review of the approaches, methodology, and interpretation of traditional closed-vocabulary text analysis from the specific perspective of multicultural and multilingual research. Attention is paid to three fundamental challenges: (1) the specifics of language and culture, (2) the levels of language analysis in question and the terminology used, and (3) the context of the use of specific tools and methods. The article ends with a discussion of possible adjustments and extensions to methods and outlines further perspectives and desiderata for conducting cross-language research in psychology.

## Challenges in Cross-Language Psychological Text Analysis

Over the last two decades, research on psychological aspects of natural word use (Pennebaker et al., 2003; Ramírez-Esparza et al., 2008; Harley, 2013) has provided an impressive bedrock of scientific findings. Most of this research has been carried out using closed-vocabulary approaches, methods based on assigning words within a target text document to categories of a predefined word dictionary (Eichstaedt et al., 2020). Semantic and grammatical features of word use have been identified as psychological markers of personal speaker characteristics, for example, gender and age (Biber, 1991; Mehl and Pennebaker, 2003; Newman et al., 2008), personality characteristics (Tausczik and Pennebaker, 2010; Yarkoni, 2010; Gill and Oberlander, 2019), social characteristics (Berry et al., 1997; Avolio and Gardner, 2005; Dino et al., 2009; Kacewicz et al., 2014), emotions (Brewer and Gardner, 1996; Pennebaker and Lay, 2002; Newman et al., 2008), and health (Ramírez-Esparza et al., 2008; Demjén, 2014). The research has so far mostly been conducted within an explanation framework, but is now also increasingly used for prediction purposes (Yarkoni and Westfall, 2017; Johannßen and Biemann, 2018).

The large number of existing studies speaks to the high relevance of this research, both in terms of establishing consensus between studies and in revealing relationships with other variables as support for concurrent validity with the results of established measures. However, recent studies have also raised important questions about the generalizability of existing findings beyond the original context of investigation, which has highlighted potential constraints on their validity in different languages and cultures (Garimella et al., 2016; Basnight-Brown and Altarriba, 2018; Jackson et al., 2019; Sánchez-Rada and Iglesias, 2019; Chen et al., 2020; Thompson et al., 2020; Dudãu and Sava, 2021). The results of the studies also indicate that the comparison and psychological interpretation of linguistic phenomena between different cultures and languages is subject to several fundamental challenges.

### Language and Culture in Question

The first challenge concerns the choice of the language and culture in which the texts are analyzed and interpreted. Currently, the vast majority of psychological language research is based on English, which dominates contemporary science as a lingua franca (Meneghini and Packer, 2007; Seidlhofer, 2011). The preference of research in English is understandable—English is a global language (e.g., the most used language of international communication, information technology, and on the Internet) (Internet Users by Language, 2021), English is the consensual language of academic discourse and, as such, it has a broad research base (Johnson, 2009). Nevertheless, the number of English native speakers (approx. 360–400 million) (König and van der Auwera, 2002), is a small fraction of the world’s population. There are approximately 6,900 languages spoken today, of which 347 have more than 1 million speakers (Bender, 2011).

Although it may seem that languages are rather similar to each other, in many cases they exhibit substantial phonological, morphosyntactic, and semantic structural differences. In other words, they operate with different linguistic building blocks, structures, and relations to communicate equivalent ideas (Haspelmath, 2020). As an example, we can describe the variance that exists in even such a basic classification as content (lexical) vs. function (grammatical) words (Corver and van Riemsdijk, 2001). Although most languages allow a relatively clear distinction between these two types, this is not the default for all languages (Asher and van de Cruys, 2018). For example, in indigenous North American languages, the words “sit,” “stand,” and “lie,” considered content words in English, appear as both content and function words (Hieber, 2020). Moreover, many word classes (parts of speech) are not present in some languages (e.g., adjectives are not present in Galela language) (Rijkhoff, 2011). Such differences exist at all levels of language (i.e., language domains, parts of grammar) and further examples will be given below.

In addition to differences between individual languages, differences between cultures using the same language should also be mentioned. As an example, we can use English, which is currently the official language in at least 58 countries (List of Countries Where English Is an Official Language – GLOBED, 2019). Not surprisingly, the use of English shows a number of variations across these cultures. The variations are most often manifested at the level of pragmatics (e.g., accentuated manifestations of egalitarianism in western Anglophone cultures compared to more pronounced patterns of respect in Asian and Polynesian Anglophone cultures) (Thomas and Thomas, 1994), but also at the level of semantics—in understanding the meaning of words (e.g., the word “old” is usually more semantically related to “age” in Australian English and to the “past” in American English) (Garimella et al., 2016). Other aspects also contribute to language variation, such as dialects or the specific use of English by non-native speakers (Wolfram and Friday, 1997; Yano, 2006). Considering that languages show such variability at both intra-lingual and inter-lingual levels, and function differently in many aspects, this may raise the question of the adequacy of single-language results (or single-culture results) that are often implicitly assumed to be broadly applicable (Wierzbicka, 2013).

### Definition of Levels and Variables of Language Analysis

The second challenge consists of the definition of the level of language (language domain, area of linguistic analysis) we focus on, the terminology used, and the variables in question. In research on the psychology of word use, terminology is often not set in accordance with traditional taxonomy in linguistics and does not adequately reflect interlingual differences. Instead of distinguishing language levels (domains) in dimensions which are more universal and established among linguists, e.g., morphology, syntax, semantics, lexicology, etc. (Hickey, n.d.; Mereu, 1999; Kornfilt, 2020), the focus of the analysis is often described in eclectic ways, based on the specifics of the language in question. For example, English is a language that has a relatively poor morphology compared to other Indo-European languages (Vannest et al., 2002; Milizia, 2020), and the level of morphology is therefore often integrated into a group of diverse variables or is replaced by other concepts. A common example is the sorting of language features into fuzzy categories such as “Linguistic Dimensions” (covering word classes and morphology), “Other Grammar” (covering word classes and both morphology and syntax), and “Psychological Processes” (covering semantics, morphology, syntax, and pragmatics together) in the LIWC2015 program (Pennebaker et al., 2015) (note: this method is described in more detail below). In fact, each of these categories includes strictly linguistic dimensions (variables), only in different configurations.

Another example is the differentiation between ‘language content’ (content of communication, that is, what is communicated/told, that usually covers lexical and semantic level) and ‘language style’ (the way the content is conveyed, that is, how the author is communicating, theoretically covering all levels of analysis, including morphology) (Ireland and Pennebaker, 2010). The assumption that language content and style can be unambiguously distinguished at the level of individual variables is questionable, since the definition of words as “content” (e.g., nouns, verbs) or “stylistic” (e.g., pronouns and prepositions) varies considerably between languages (Corver and van Riemsdijk, 2001; Asher and van de Cruys, 2018; Hieber, 2020). Even the most general distinction between function words and content words in one language captures rather a continuum, where prototypical function words and content words appear at opposite ends (Osborne and Gerdes, 2019). In summary, although these conceptual or effectively metaphorical distinctions have proven theoretically generative and practically useful, they can significantly limit the possibilities of cross-language comparison.

The unclear taxonomy and exclusive, domain-specific terminological definition bring with them complications both at the level of interdisciplinary cooperation (e.g., among psychologists and linguists) and at the level of intercultural research (Sonneveld and Loening, 1993). For languages that are relatively close in their structure, the discrepancy in classification may not be pronounced, but when distant languages are studied and compared, substantial differences can arise. The taxonomy of words and their functions is non-trivially language-specific, with different languages providing different classifications of language content and style (Nivre et al., 2016; Kirov et al., 2020). In some languages, the same grammatical relationship is expressed morphologically, in others through function words, while some languages do not mark this information at all (e.g., in grammatical tense or definiteness) (Osborne and Gerdes, 2019; Universal Dependencies: Syntax, 2021).

For example, many locatives are marked by prepositions in English (e.g., “in,” “by,” “to,” “from”), while in Finnish they appear as morphological case-inflections (e.g., “-ssA,” “-llA,” “-lle,” “-stA,” “-ltA”). Furthermore, possessives and adverbials can be marked morphologically in Finnish (e.g., “-ni”—“my,” “-si”—“your”), but in English they appear as separate words, thus a word form like “auto-i/ssa/ni/kin” (“also in my cars”) with stem and four subsequent suffixes would need four separate words in English (Vannest et al., 2002). The Czech language provides another example of the interconnection between language content and style. It also works with a wide range of grammatical suffixes that change paradigmatic and grammatical classification, e.g., the word “uč” (“teach!”) with suffixes “-it” (“to teach”) “-el” (“teacher”) “-ova/á” (“of teacher”) “-ní” (“teaching”) “-čko” (“little teaching”), where each of the suffixes can changes the inflection and/or semantic nature of the word (Rusínová, 2020). Therefore, a text analysis approach that counts and processes such linguistic units as stand-alone words (Pennebaker and King, 1999; Pennebaker et al., 2014) is inherently limited and potentially biased.

### Approaches and Methods in Question

The third challenge concerns specifics around the commonly employed text analytic approaches and methods. Many methods were primarily designed for the processing of a specific language, or even a specific type of communication (i.e., genre or register), and their use in cross-language research can therefore result in methodological and interpretive difficulties. In this regard, the current approaches to quantitative text analysis, based on lexical and semantic levels of analysis (treating words/tokens as lexical units within a certain semantic field) (Cruse et al., 1986), can be divided into two main groups—closed-vocabulary approaches and open-vocabulary approaches (Schwartz et al., 2013b). Closed-vocabulary approaches operate from “top down” and assign words from a target text to psychologic categories within a specific and fixed dictionary (e.g., a dictionary of emotional words that covers categories of positive and negative emotion categories). This procedure is also referred to as the word-count approach (Schwartz et al., 2013a; Iliev et al., 2015; Kennedy et al., 2021). The result of the analysis is usually the (normalized) frequency within which references to these categories occur in a given text (Eichstaedt et al., 2020).

Compared to that, open-vocabulary approaches operate from “bottom-up” (data-driven), that is, based on language (text) as such. Algorithms identify related clusters of units (lexical units or elements, for example, punctuation) that naturally occur (and co-occur) within a large set of texts and find lexical and semantic patterns that appear (and appear together) in the data (Park et al., 2015; McAuliffe et al., 2020). Both approaches have their pros and cons; as stated by Eichstaedt et al., “Closed-vocabulary approaches can be rigid, while open-vocabulary approaches can be sensitive to idiosyncrasies of the dataset and the modeler’s choices about parameters. Closed-vocabulary approaches are more reproducible but inflexible, where open approaches are more flexible but can vary across datasets” (p. 77) (Eichstaedt et al., 2020). Given the historical dominance of word-count approaches, the following section focuses in detail on closed-vocabulary analysis.

## Closed-Vocabulary Approaches in Cross-Cultural Research

In terms of the number of published studies, closed-vocabulary approaches still dominate by far the field of psychology of word use. There are many reasons for their preference, for example, their implementation exacts little technical demands (training of the AI, development of algorithms, etc.), they allow relatively uncomplicated interpretation of the results, and they also do not require large datasets to perform the analysis (Eichstaedt et al., 2020; Sharir et al., 2020). Over the last six decades, a number of tools have been developed, e.g., General Inquirer (Stone et al., 1962), DICTION (Hart, 2001), Linguistic Inquiry and Word Count (LIWC) (Pennebaker et al., 2015), Affective Norms for English Words (ANEW) (Bradley and Lang, 1999), SentiStrength (Thelwall et al., 2010), SentiWordNet (Baccianella et al., 2010), OpinionFinder (Wilson et al., 2005), Regressive Imagery Dictionary (Martindale, 1973), TAS/C (Mergenthaler and Bucci, 1999), Gottschalk-Gleser Scales (Gottschalk et al., 1969), or Psychiatric Content Analysis and Diagnosis (PCAD) (Gottschalk, 2000).

Most of these methods are primarily focused on the level of lexical semantics, that is, on searching for words with specific semantic loading. The analyzed text is usually compared with a predefined dictionary that contains words that represent a concept (e.g., religion words) or a psychological state (e.g., positive emotion words). For example, the concept of ‘satisfaction’ in DICTION is represented by words such as “cheerful,” “smile,” or “celebrating” (Hart and Carroll, 2011). Leaving aside the question of the validity of the semantic categories in the dictionary itself (cf. Garten et al., 2018), there are several issues that closed vocabulary analysis has to deal with. A common problem is the interpretation of lexical ambiguity and the meaning of words in different contexts (Hogenraad, 2018). A typical example in English are contronyms or polysemous words such as “fine” (signifying both pleasant and a penalty), “mean” (signifying both bad and average), and “crazy” (signifying both excitement and mental illness). The risk of misinterpretation (misclassification) can be reduced by, e.g., removing or replacing ambiguous words from the dictionary (Schwartz et al., 2013a). However, such a procedure almost necessarily also reduces the sensitivity of the semantic category, and thus the precision of the analysis.

### Level of Lexical Semantics in Cross-Language Adaptation

If we focus on cross-language adaptation of closed-vocabulary methods, it should be emphasized that these tools are naturally based on the specifics of the source (original) language for which they were developed, most often English [see Mehl (2006)]. Therefore, adapting such dictionaries to other languages is often a complicated and time-consuming process that faces a series of additional challenges (Bjekić et al., 2014; Dudãu and Sava, 2020; Boot, 2021). First, the methods are most often based on the original cultural and linguistic structure rather than the target culture or language, that is on the imposed-etic approach (Berry et al., 1997). This strategy can lead, among others, to the risk of reductionism or misinterpretation of results, for example, when constructs (variables/categories) do not exist, are not equivalent, or function differently in the original and target language (Church and Katigbak, 1989). Languages often have unique words that are difficult to express in other languages (e.g., words like “toska” in Russian, “jamani” in Swahili or “saudade” in Portuguese). Furthermore, even for words that seem easy to translate, their meaning may shift, e.g., in English, the word “anger” is mainly related to wrath, irateness or rage, while in the Nakh-Daghestanian languages, it is closer to envy and in the Austronesian languages more closely associated with pride (Jackson et al., 2019).

Let us add that semantic changes are not a matter of cross-language comparison only, but they can also occur naturally within one language, such as in different historical stages of a language (Vanhove, 2008; Riemer, 2016; Garten et al., 2018).

Second, the possibility of estimating possible shortcomings of dictionary adaptation can be problematic, since the degree of equivalence varies not only across language features (some words are more cross-linguistically and cross-culturally comparable than others) (Biber, 2014), but also across different communication contexts (Daems et al., 2013; Biber and Conrad, 2019; Dudãu and Sava, 2020). For example, the meaning and use of the English word “hump” vary both between English speaking cultures and between situational contexts (e.g., in British English it can refer to an emotional state, in American English it can refer to a vigorous effort, depending on the context in which it can be perceived as vulgar). In some languages, the influence of the context is crucial for the word interpretation and classification, such as in Czech, where sociolinguistic situation (inter-lingual variation) borders on diglossia (Bermel, 2014). Thus, we can assume that dictionaries validated only in a certain communication context (e.g., academic essays) will not be sufficiently effective in another context (e.g., informal conversations).

The topic of comparability of language variables (words, units, features) across languages is discussed in a number of studies. Although many of them have revealed a high degree of similarity in the results of cross-language analysis (Ramírez-Esparza et al., 2008; Windsor et al., 2019; Vivas et al., 2020), there is increasing evidence pointing to significant differences in lexical and semantic functioning across more distant languages. In the study by Thompson et al. (2020), published in *Nature Human Behavior*, the authors analyzed semantic alignment (neighborhood) for 1,010 meanings in 41 languages using distributed semantic vectors derived from multilingual natural language corpora. While some words within semantic domains with a high internal structure were more closely aligned across languages—especially quantity, time, and kinship (e.g., “four,” “day,” and “son”), words denoting basic actions, motion, emotions and values (e.g., “blow,” “move,” and “praise”) aligned much less closely. In terms of semantic alignment by parts of speech (word classes), the highest alignment was found in numerals, while other parts of speech were much less aligned (e.g., prepositions were the least aligned). Thus, this study critically questions the idea of widely comparable word meanings across languages, at least from a cross-cultural universalist perspective (Kim et al., 2000).

Another study, published in *Science*, examined nearly 2,500 languages to determine the degree of similarity in linguistic networks of 24 emotion terms (Jackson et al., 2019). The study also revealed a large variability in the meaning of emotion words across cultures. For example, some Austronesian languages colexifies the concepts of “pity” and “love,” which may index a more positive conceptualization of “pity” compared to other languages. Another example concerns the connotation of “fear,” which is more associated with “grief” and “regret” in Tai-Kadai compared to other languages. As the authors show, the similarity of emotion terms could be predicted based on the geographic proximity of the languages, their hedonic valence, and the physiological arousal they evoke. Given the central role of emotion words, and more broadly sentiment analysis, in the field of language analysis, this study has clear implications for cross-language analysis, particularly when comparing distant cultures and languages.

Finally, cultural differences in language use were also documented in a study that focused only on English. Garimella et al. (2016) described the differences between Australia and the United States based on the words they used frequently in their online writings. The results indicated that there are significant differences in the way these words are used in the two cultures, reflecting cultural idiosyncrasies in word use. For example, the adjective “human” is more related to human rights in the Australian context, but more to life and love in the United States context (Garimella et al., 2016). From our point of view, these studies provide important insights: although languages are similar in many ways and they certainly share universal bases, the degree of similarity varies depending on cultural and geographical specifics.

## The Linguistic Inquiry and Word Count Program as an Example

So far, we have focused on the analysis on the lexical semantics level only—this level is also common to all closed vocabulary approaches mentioned above. However, one of the methods, the LIWC program, is exceptional in this respect—besides traditional semantic categories (social words, emotion words, etc.), it provides an additional analysis of morphology and syntax features. Therefore, LIWC therefore serves well to illustrate the potentials and pitfalls of cross-linguistic adaptation of the closed vocabulary method in the context of multiple language levels (domains).

Linguistic inquiry and word count (Pennebaker et al., 2015) is currently the most widely used text analysis method in the social sciences. At the time of writing this article, 781 records were available on the Web of Science that contained “LIWC” or “Linguistic Inquiry and Word Count” as the topic, and more than twenty thousand records are listed on Google Scholar. In its current version, LIWC2015, the program offers an intuitive user interface and provides a simple and clear output of the results (Pennebaker et al., 2015), including a range of comparison possibilities (Chen et al., 2020). LIWC dictionaries have been translated and adapted into multiple languages, including Spanish (Ramírez-Esparza et al., 2007), French (Piolat et al., 2011), German (Wolf et al., 2008; Meier et al., 2019), Dutch (Boot et al., 2017; Van Wissen and Boot, 2017), Brazilian-Portuguese (Balage Filho et al., 2013; Carvalho et al., 2019), Chinese (Huang et al., 2012), Serbian (Bjekić et al., 2014), Italian (Agosti and Rellini, 2007), Russian (Kailer and Chung, 2007), Arabic (Hayeri, 2014), Japanese (Shibata et al., 2016), and Romanian (Dudãu and Sava, 2020).

English LIWC2015 works with approximately 90 features grouped into 4 domains: “Summary Language Variables” (general text descriptors and lexical variables, including one syntactic variable “words per sentence”), “Linguistic Dimensions” (containing summary variables, word classes variables, and morphological variables, e.g., “total function words “, “articles,” “1st person singular,” and “negations”), “Other Grammar” (containing word classes variables, and both morphological and syntactic variables, “numbers,” “comparisons,” and “interrogatives”), and “Psychological Processes” (containing semantic variables and other variables, e.g., “sadness,” “non-fluencies,” and “causation”) (Pennebaker et al., 2015). In terms of the analytic procedure, LIWC operates on relatively simple principles. LIWC uses its own dictionary to simply identify and label the corresponding words in the analyzed text—*via* word-count. Pre-processing in LIWC includes only basic segmentation and requires additional manual tagging (e.g., for specific ambiguous filler words, e.g., “well,” “like,” or non-fluencies, e.g., “you know”). More advanced NLP procedures, on the other hand, use pre-trained models and perform a sequence of “cleaning” processes in such tasks (e.g., Rayson, 2009; Manning et al., 2014), e.g., part of speech disambiguation and tagging, lemmatization, or parsing (Straka and Straková, 2017).

Several strategies have been used to adapt the LIWC dictionary to other languages (Boot, 2021). These include the supervised translation of the English dictionary word by word (Bjekić et al., 2014; Dudãu and Sava, 2020), the use of the existing word corpora and their assignment to corresponding LIWC categories (Andrei, 2014) or as an enrichment of LIWC categories (Gao et al., 2013; Meier et al., 2019), the use of dictionaries in closely related languages (Massó et al., 2013), the modification of the older version of the dictionary (Zijlstra et al., 2004), or adapting the original dictionary *via* machine translation (Van Wissen and Boot, 2017). The various LIWC languages differ significantly in the number of words contained in the dictionary. For example, the Romanian LIWC dictionary (Ro-LIWC2015) contains 47,825 entries compared to the English LIWC2015 dictionary with 6,549 entries (including words, word stems, and emoticons; cf. LIWC2007 contains 4,500 words, and LIWC2001 contains 2,300 words). The average proportion of words identified (labeled) by LIWC also varies considerably across the different LIWC language dictionaries, for example 87% in English (LIWC2015; cf. 82% in LIWC2007), 88% in German (DE-LIWC2015; cf. 70% in LIWC2001), 70% in Dutch, 54% in French, 66% in Spanish, 70% in Serbian, and 67% in Romanian (Bjekić et al., 2014; Dudãu and Sava, 2020), speaking to the fact that the LIWC approach likely yields differential sensitivity across different languages.

### Analysis of Non-semantic Levels of Language

The translation and adaptation process faces most of the issues described above. Here, however, the analysis deals also with additional challenges, connected to level of morphology and syntax of the target languages, for example the pronoun-drop phenomenon (in some languages, users very frequently omit pronouns, particularly in their subject positions; e.g., “tengo hambre” in Spanish dropping the first-person singular pronoun “yo”) (Świątek, 2012), grammatical classification (e.g., pronominal adverbs in Dutch, that combine pronouns/adverbs with prepositions—“we doken erin” which replaces “we doken in het”—“we dived into it”), grammatical restrictions (some linguistic features are restricted to particular languages, see below), with case sensitivity problems (LIWC is not case-sensitive which makes it difficult to process certain words, e.g., the German word “Sie” which, if capitalized, serves as formal second person singular or plural pronoun and, when not capitalized, serves as third person plural pronoun), and the above mentioned ambiguity (including, if the capitalized word appears at the beginning of a sentence) (Boot, 2021).

Although some shortcomings of the dictionary translation approach can be partially overcome (e.g., by removing words from the dictionary, adding new words and phrases, or with data pre-processing), they still increase the risk of reduced sensitivity and validity, especially in its reliability and comparability to the original method. As already mentioned, this applies particularly to languages with a grammatical structure more distant from English. For example, due to the grammatical structure of Serbian (a Slavic language), the category of verbs had to be substantially modified, and the category of articles had to be removed completely (Bjekić et al., 2014). Many adjustments were also made in the Romanian adaptation, for example in verb tense, grammatical gender, or diacritics processing (Dudãu and Sava, 2020). To sum up, every translation of the LIWC dictionary involves many decisions about which entries (words or categories) should be kept, dropped, or added, and each decision is necessarily a trade-off between computational feasibility and linguistic accuracy (Dudãu and Sava, 2021).

### Cross-Language Evaluation of Linguistic Inquiry and Word Count

The extent to which language specifics and LIWC adjustments affect the quality of adaptation is difficult to evaluate, as the studies differ in many aspects. Some studies do not report psychometric validation information for their dictionaries (e.g., Arabic, Turkish, or Russian), while others provide only indirect evidence (Balage Filho et al., 2013). In several studies, equivalence estimates are presented as a general indicator of the quality of adaptation. Equivalence is usually estimated *via* correlation coefficients between the adapted version of LIWC and the English original. If we focus on four major studies, the authors report an average correlation of adapted LIWC and English LIWC as *r* = 0.67 for German based on *N* = 5,544/6,463 texts in German/English (Europarl corpora and transcriptions of TED Talks transcriptions), *r* = 0.65 for Spanish (*N* = 83 texts in Spanish/English; various Internet sources), *r* = 0.65 for Serbian (*N* = 141 texts in Serbian/English; scientific abstracts, newspapers and movie subtitles), and *r* = 0.52 for Romanian (*N* = 35 books of contemporary literature in Romanian/English) (Ramírez-Esparza et al., 2007; Bjekić et al., 2014; Meier et al., 2019; Dudãu and Sava, 2020).

Although the average values of the correlations can be considered satisfactory, upon closer inspection, they vary widely between categories and levels of analysis, especially in morphology and semantics. For example, in the Romanian LIWC, most correlations of non-semantic categories are non-significant (11 of 18 categories). Significant results were found in the category “Pronouns” in the first person (singular 0.93, plural 0.92) and in the third person singular (0.66, plural non-significant), in the category “Other Function Words” in conjunctions (0.37) and negations (0.53) and in the category “Other Grammar” in interrogatives (0.58) and quantifiers (0.66) (Dudãu and Sava, 2020). Considering these results and the average proportion of total words identified in the Romanian LIWC (only 67% words were labeled), we must conclude that the Romanian LIWC appears not effective enough for the comparable analysis of non-semantic (grammatic) categories, even though its dictionary is seven times bigger than the English original (Romanian: 47,825 entries; English: 6,549 entries; Dudãu and Sava, 2020).

Another issue concerns the specificity of text samples on which validity and equivalence tests were performed. In this sense, the communication context (text type, genre, register) is an important factor that can produce substantial variation both in the frequency of language features and in the associations with other variables, especially psychological ones (Pennebaker et al., 2007; Daems et al., 2013; Haider and Palmer, 2017; Biber and Conrad, 2019; Kučera et al., 2020; Dudãu and Sava, 2021). Differences in the sensitivity of LIWC for detecting psychological markers in different types of text (English only), were shown in the meta-analysis of Chen et al. (2020), in which, for example, the strength of the relationship between extraversion and positive emotion words varied significantly and substantially across communication contexts (e.g., asynchronous/synchronous and public/private communication). Thus, if only one type of communication is used (e.g., only written language), it is difficult to estimate to what extent the translated dictionary has comparable validity for, for example, spoken communication. Moreover, it is possible to assume that the language variation is related to multiple factors, not only to the type of text, but also to, for example, sociodemographic characteristics of speakers (Stuart-Smith and Timmins, 2010), as well as to discourse domain and language itself (Biber, 2014).

The above-mentioned challenges have implications not only for the adaptation of closed-vocabulary methods to other languages, but for the field of psychology of word use more broadly. Due to the predominant interest of research in the English language, psychological language markers are often implicitly presented in studies as relatively universal, generalizable at least to English-speaking cultures (Chung and Pennebaker, 2018). In many classical studies, for example, frequent use of first-person singular pronouns has emerged as a marker of negative emotionality (Pennebaker and King, 1999; Pennebaker et al., 2003; Oberlander and Gill, 2006; Gill et al., 2009; Yarkoni, 2010; Qiu et al., 2012). However, subsequent research in other languages and on other samples relativizes this relationship (Mehl et al., 2012; Bjekić et al., 2014; Holtzman et al., 2019; Kučera et al., 2020, 2021). Given the lack of cross-language and cross-cultural studies, the original assumption of generalizability is understandable. However, considering recent studies, the previous conjectures need to be corrected for regarding the culture, language, and communication contexts and samples in which the relationships emerged. If the different functioning of words in other languages and cultures is not sufficiently described, many generalizations may be biased or misrepresented as a result.

## Dealing With Closed-Vocabulary Cross-Language Analysis

Although the issues raised above may raise pessimism regarding the possibilities of closed-vocabulary approaches in cross-language research, we believe that most challenges can (and need to be) overcome, at least to some extent. Closed-vocabulary approaches offer, in contrast to open-vocabulary approaches, several advantages that are important for psychological research. The categories they work with can be intuitively labeled and (and facilitate interpretation, explanation, testing, and accumulation and transfer of results (e.g., into other languages and contexts) (Kennedy et al., 2021). Even if traditional methods are replaced by new technologies (e.g., AI), the demand for interpretations of phenomena based on intuitive categories (e.g., representing variables using established psychological concepts) is bound to survive. In the rest of the article, we therefore focus on suggestions that support the effective use of closed-vocabulary approaches in multilingual and multicultural setting.

### Dealing With Language and Culture

The first challenge we discussed was the language and culture on which the analysis is based and the degree of its similarity to other languages and cultures. To build on the previous arguments, text analysis methods likely provide more different results the further apart studied languages and cultures are, not only because of the methodological differences in analysis, but also because of the specifics of the languages and cultures themselves. As a parallel, we reference the issues concerning the use of Big Five personality questionnaires across cultures (the most widely used method for assessing personality characteristics), which outside of western, educated, industrialized, rich and democratic (WEIRD) populations shows serious limitations and low validity for measuring the domain of basic personality traits (Laajaj et al., 2019). In the same way, striving for better explanations of cross-linguistic variation requires employing the power of cross-cultural comparisons to describe the variation and similarity (Barrett, 2020)—the methodology must be linked to more principled sampling, both at the level of speakers (e.g., representative sample of speakers in a given culture or at least a sample corrected for imbalances) and texts (e.g., to acquire the texts with regard to their ability to be representative for selected communication contexts).

Since the cross-language comparison based on texts from the entire communication spectrum would be difficult to implement, it is necessary to choose specific types of communication (i.e., registers, and genres) to be analyzed. Leaving aside their ease of availability to the researcher, the focus should be on types of text that show a certain degree of cross-language universality. In this regard, existing cross-linguistic studies on register variation can provide important information in this regard. For example, Biber’s research finds two language dimensions (i.e., constellations of linguistic features that typically co-occur in texts) that could be considered relatively (although not absolutely) universal: (1) “clausal/oral” discourse vs. “phrasal/literate” discourse, and (2) “narrative” vs. “non-narrative” discourse (Biber, 2014). The first dimension linguistically comprises typical grammatical features (e.g., verb and pronoun classes) and is based functionally on a distinction between a personal/involved focus and informational focus (e.g., private speech vs. academic writing as prototypic genres). The second, narrative dimension, consists of different sets of features (e.g., human nouns and past tense verbs), and typically appears in fictional stories, personal narratives, or folk tales. These general patterns have emerged from different studies of languages other than English, for example, Spanish, Brazilian Portuguese, Nukulaelae Tuvaluan, Korean, Somali, Taiwanese, Czech, and Dagbani (Biber, 2014).

From the point of view of cross-language comparison, it is therefore recommended to choose text types that are at least somewhat comparable on these two dimensions to ensure maximum (in the sense of as much as reasonably possible) comparability. If the selection of texts cannot be made by dimensions defined *ex ante* (e.g., if the texts have already been collected), it is also possible to subject the texts to *ex post* dimensional analysis *via* multi-dimensional analysis (MDA), an approach that identifies co-occurrence patterns of linguistic features based on the factor analysis (Biber, 1991). Through MDA, it is possible to describe different texts in terms of their similarity in dimensional structure. However, MDA is currently only available for a limited number of languages (in addition to the languages listed above for Scottish Gaelic and written Chinese) (Sardinha and Pinto, 2019).

### Dealing With Levels of Analysis and Language Variables

The second challenge concerns the terminology and language level (domain) that is the subject of the analysis. Since the definition of language variables based on the specifics of one language only is problematic, it is necessary to work with variables that have common characteristics and to categorize them in a more clearly defined system. The issue of universal classification has been addressed in a number of studies, both theoretically and practically (Hasselgård, 2013). If we are to build on newer approaches, two of the available linguistic frameworks can serve as an example to follow, the Universal Dependencies (UD) and the Universal Morphology (UniMorph) projects (Nivre et al., 2016; McCarthy et al., 2020). Both frameworks focus on the annotation of human language and connect many fields of contemporary linguistics (Osborne and Gerdes, 2019; de Marneffe et al., 2021). In both frameworks, morphology (including part of speech) and syntax are considered the most principal non-semantic levels of language analysis in the taxonomies.

Universal Dependencies^1^ is a framework for annotation of grammar across different human languages, currently available for 122 languages with 33 more in preparation (Universal Dependencies, 2021). Morphological variables of UD include, for example, the categories of part of speech and lexical and inflectional features (e.g., pronominal type and degree of comparison), and syntactic variables include cover dependency relations between words (relations between a syntactic head and a subordinate element, e.g., multiple determiners attached to the head noun).

The UniMorph project^2^ has similar goals as UD and provides normalized morphological paradigms for diverse world languages, especially low-resource languages with inflectional morphology. The schema of UniMorph comprises 23 dimensions of meaning (e.g., person, number, tense, and case) and over 212 features (for the dimension of case, e.g., ablative, absolutive, accusative, etc.) (Sylak-Glassman, 2016; McCarthy et al., 2020).

If we consider Universal Dependencies and the Universal Morphology frameworks from the perspective of cross-language research, i.e., when comparing multiple languages analyses, a comment needs to be added to the number and applicability of linguistic variables. Since the set of linguistic features (categories, dimensions) we can work with is entirely dependent on properties of languages in question, it is necessary to identify features that are shared between these languages—i.e., identically labeled in UD or UniMorph. For example, if we compare the results of UD text analysis in English and Spanish, we can only work with 13 English features, which are shared with Spanish (e.g., degree, gender, person, polarity; see English ParTUT and Spanish AnCora treebanks; Universal Dependencies, 2021). However, UD in Spanish offers more linguistic features (23 features in total), and we can use these “non-English” variables, e.g., in a further comparison with another language.

To sum up, the frameworks provide useful tools, and they can serve as a starting point for better classification and (re-)definition of language variables for the purposes of cross-language psychological analyses. In addition, Universal Dependencies Tools are open-source software, so they are available for free.

### Dealing With Cross-Language Adaptation of Methods

The third challenge is related to current approaches to text analysis and their methods. In terms of cross-language use of semantically based closed-vocabulary approaches, research should focus primarily on identifying and covering the semantic specifics and functioning of words in different languages, not just on translating the text into the language of analysis. Studies that describe the semantic alignment of words across different languages and contexts could help here (Garimella et al., 2016; Jackson et al., 2019; Thompson et al., 2020). For both semantic and morphological analysis, several procedures can be used to increase the comparability of the analyses. For example, it is possible to use statistical adjustments proposed by Dudãu and Sava—to employ multilevel analysis with language as the level 2 covariate (especially when text input is available in relatively different languages) or to perform within-language standardization to attenuate the language particularities that could affect the investigation in the multilingual setting.

For example, Brazilian Portuguese probably has linguistic particularities in the use of third-person singular (e.g., in personal pronouns and possessives with a higher degree of inflection), which can cause inconsistencies in cross-language comparisons (Carvalho et al., 2019). To avoid the lack of equivalence between results of analyses in different languages, it is possible to perform within-language standardization, i.e., use the mean and standard deviation of the third person singular variable as the reference parameters for rescaling the values. As the authors state, when comparing the four LIWC language adaptations (English, Dutch, Brazilian Portuguese, and Romanian), the unadjusted calculations show little sign of cross-language equivalence compared to the situation where language specificities are considered, that is, *via* within-language standardization (Dudãu and Sava, 2021).

Another way to reduce the difficulties of adapting closed-vocabulary methods and subsequent cross-language comparison is to use machine translation. Two basic approaches are the “translated dictionary” approach, and the “translated text” approach. The first one consists of automatic translation of entries (usually word by word) from the original dictionary (e.g., English) into the target language. This creates a new dictionary in the target language, which is used to perform analyses in this particular language (e.g., the Danish version of LIWC) (Boot et al., 2017; Van Wissen and Boot, 2017). The second approach consists of translating the analyzed text into the language in which the original method works (e.g., English) and then in performing the analysis with the original method. This approach seems to be effective and straightforward in many ways—it makes the analysis tool accessible to languages for which it has not yet been adapted, and reduces errors associated with the translation process and adaptation of the dictionary into another language. The efficiency of MT systems (e.g., Google Translate) is proving to be very high also in terms of syntax and stylistics and recent studies show that this “translated text” approach outperforms the traditional word-by-word “translated dictionary” approach (Windsor et al., 2019; Araújo et al., 2020; Boot, 2021), for example, in measures of equivalence of Dutch, German, and Spanish language analyses (Boot, 2021).

### Dealing With Methods Based on Machine Learning

Finally, acknowledging where the field is heading, we would like to comment on questions around new technologies in psychological text analysis more generally. The use of artificial intelligence (AI), machine learning (ML), and machine translation (MT) is already closely related to many aspects of text analysis, for example, within open-vocabulary approaches (Eichstaedt et al., 2020). Undoubtedly, modern technologies offer enormous potential based on the performance and sophistication of up-to-date computational systems, but also raise fundamental questions about methods of data processing, their supervision, and interpretation of results (Mønsted et al., 2018; Stachl et al., 2020).

The ML and MT methods allow us to expand the spectrum of observed variables and at the same time effectively predict their relationships. However, from the perspective of our paper, their disadvantage is the problematic interpretation of the analytical processes itself, i.e., the so-called black box problem (Castelvecchi, 2016). For example, it is possible to train AI on a large number of texts to effectively recognize the specific characteristics of speakers (and then, e.g., allow the AI to predict them), but it is difficult to get clearer information on what procedures and variables (features) are involved in the process (Zednik, 2019). AI is thus more of a promising method for predicting relationships, rather than a method that provides their explanation and deeper insight (Yarkoni and Westfall, 2017).

It is not within the scope of this article to discuss all aspects of ML/MT utilization; however, we would like to focus on one issue that we consider particularly important in relation to cross-language research and the use of closed-vocabulary analysis in psychology. These are the quality and complexity of the training data, especially in the context of different languages and different types of communication.

Successful use of ML depends to a large extent on the data on which the system is trained, both in terms of quantity and quality (Ehrlinger et al., 2019). Regarding the number of training texts, a general rule of thumb is that more data usually means higher effectiveness of the system (Baeza-Yates and Liaghat, 2017). In terms of data quality, the situation is much less clear. In addition to routine data quality controls (e.g., cleaning dataset from irrelevant texts), the nature of texts should also be considered, especially at the level of the type of communication that is the subject of the ML training (Smith et al., 2013; Modaresi et al., 2016; Medvedeva et al., 2017; Ott et al., 2018). For example, several studies have shown that current electronic communication is dominated by the so-called “electronic/internet discourse” (e-discourse), which takes the form of semi-speech (between speaking and writing) (Abusa’aleek, 2015). This e-discourse has its own features such as unconventional spelling and combinations of visual and textual elements (Lyddy et al., 2014; Pam, 2020).

Following this concept, we can assume that if ML is, say, trained primarily on parallel corpora of formal written communication (e.g., press releases or parliament transcripts in two or more languages), its effectiveness for processing (translating) the e-discourse or other more specific communication might be noticeably reduced, and vice versa (Koehn and Knowles, 2017; Søgaard et al., 2018). Increased error rates for certain types of text (styles, genres, registers) have been described for systems as complex as Google Translate (Putri and Havid, 2015; Afshin and Alaeddini, 2016; Prates et al., 2018). These errors mainly concern lexical/discourse errors and style errors (note: lexical errors occur when MT translates words wrongly or does not translate them, discourse errors occur when MT could not recognize the meaning of the word in its context, and style errors occur when the word is inappropriate in a given context). In the 2016 research, error rates (based on comparison with human translation) were quantified at 5.9% for lexical/discourse errors and 8% for style errors (Afshin and Alaeddini, 2016). Higher sensitivity to errors was found in the translation of function words, especially adjectives and adverbs (Putri and Havid, 2015). In addition to these errors, problems referred to as “machine-bias” can arise. A classic example is the case of gender preference in Google Translate, that is, when Google MT exhibited a strong tendency toward male defaults (Prates et al., 2018). Although the issue was quickly handled by Google through (forced) equal representation of gender categories in translation, the underlying problem itself is not resolved that easily, since MT was probably trained on (historical) data in which the male gender is more common, which resulted in the preferred in translation. In these situations, it is therefore necessary to apply methods such as “post-editing,” i.e., the process of making corrections or amendments to automatically generated text (machine translation output) (Temizöz, 2016; Gutiérrez-Artacho et al., 2019).

The quality of MT is constantly changing with the ever-increasing training data and the participation of new technologies (e.g., automatic transcription of oral communication). At the same time, the accumulation of data facilitates the representation of more diverse types of communication and language varieties (dialects, sociolects, etc.), which contributes to solving number of problems of traditional closed-vocabulary approaches (MT is based on authentic varieties of language, not on *a priori* assumptions about their functioning). However, the increase in the amount of training data is not proportional between languages—languages that are used more often in electronic communication (especially English) provide automated systems with much more data than the so-called “low-resource/resource-poor languages” (Thuy et al., 2018). Although it is possible to apply procedures that link datasets of resource-sufficient and resource-poor languages (Impana and Kallimani, 2017), the issue of reduced comparability cannot be overlooked (Seki, 2021). The described situation is a parallel to the previously mentioned problem of disproportionate representation of certain types of communication in the ML dataset. In the application of MT in psychological research, it is therefore necessary to emphasize the need for control and documentation of the ML training process, especially when working with languages that generate fewer texts compared to the world’s most used languages, and when working with types of text that are more distant to original training data.

## Conclusion

At the beginning of our article, we stated that we are currently in a “transitional phase of research” within the field of text analysis. After more than 60 years of research on psychological aspects of word use, new technologies and methods are entering this discipline at a rapid rate. Original programs based on simple word counting are being challenged by automated machine learning systems and large-scale “big data” analyses (Gandomi and Haider, 2015) that allow for extensive cross-cultural comparisons. New technologies offer great potential, but the question is when (or whether) they will completely replace traditional techniques. It will also be important to consider to what extent the original methods can support more advanced analyses in terms of their focus, interpretation, and explanation of linguistic phenomena. In this regard, current research raises a number of questions related to the relevance of older studies, considering different language structures in different cultures and contexts of human communication (Kim et al., 2000; Jackson et al., 2019; Thompson et al., 2020).

In our critical analysis here, we focused on closed-vocabulary approaches, a relatively old method of text analysis. Nevertheless, even today, its contribution needs to be appreciated and its strengths highlighted. We would like to celebrate the ground-breaking research and many quality papers that have been published in this field over the last two decades (for all, see, e.g., Pennebaker et al., 2003). Research in Anglophone cultures has provided many excellent tools for text analysis in English, but it has also amplified universalist tendencies to adapt target languages to default methods, instead of adapting these methods to target languages and their specifics (e.g., Bjekić et al., 2014; Dudãu and Sava, 2020). Given the richness and variety among different languages, many relationships between language and psychological variables are undoubtedly reduced this way (Kim et al., 2000; Wierzbicka, 2013; Kučera, 2020).

In summary, we can state three basic considerations: (1) To further the science of the psychology of word use, it is necessary to promote close interdisciplinary cooperation, especially with the fields of linguistics, computer science, and cultural psychology. Within that, linguistics can provide a clear taxonomy of language, a background in cross-linguistic research, and useful analytic tools (e.g., MDA for dimensional text description or UD for their morpho-syntactic annotation) (Biber, 2014; de Marneffe et al., 2021). (2) If we are looking for relationships between mind, behavior, and language use, it is not possible to overlook the specifics of different languages and cultures. Although studies conducted in English are usually more accessible to both researchers and the public (e.g., given the tools available and the amount of data), it is critical to compare the results with studies in other languages and cultures in order to evaluate the generalizability of relationships and to understand their meaning more deeply (Kim et al., 2000; Wierzbicka, 2013). (3) In cross-language psychological research, all present-day methods can be used. However, it is necessary to consider their functionality in different contexts (e.g., define more universal variables and comprehend situational/cultural aspects of communication) (Biber and Conrad, 2019; Cvrček et al., 2020), and critically assess their development and use. This consideration also applies to current machine learning systems, in which the possibility of methodological supervision is usually limited (in terms of control of the analysis process) and in which the fundamental condition for their effectiveness is the quality of training data (Koehn and Knowles, 2017; Ott et al., 2018). These three points can be related to both new studies and studies already conducted, for which a review of their results could be expected.

## Data Availability Statement

The original contributions presented in the study are included in the article/supplementary material, further inquiries can be directed to the corresponding author/s.

## Author Contributions

DK: conceptualization, investigation, writing—original draft, and writing—review and editing. MM: conceptualization, supervision, writing—original draft, and writing—review and editing. Both authors contributed to the article and approved the submitted version.

## Conflict of Interest

The authors declare that the research was conducted in the absence of any commercial or financial relationships that could be construed as a potential conflict of interest.

## Publisher’s Note

All claims expressed in this article are solely those of the authors and do not necessarily represent those of their affiliated organizations, or those of the publisher, the editors and the reviewers. Any product that may be evaluated in this article, or claim that may be made by its manufacturer, is not guaranteed or endorsed by the publisher.

## Footnotes

1
universaldependencies.org

2
unimorph.github.io
